# European American Stratification in Ovarian Cancer Case Control Data: The Utility of Genome-Wide Data for Inferring Ancestry

**DOI:** 10.1371/journal.pone.0035235

**Published:** 2012-05-09

**Authors:** Paola Raska, Edwin Iversen, Ann Chen, Zhihua Chen, Brooke L. Fridley, Jennifer Permuth-Wey, Ya-Yu Tsai, Robert A. Vierkant, Ellen L. Goode, Harvey Risch, Joellen M. Schildkraut, Thomas A. Sellers, Jill Barnholtz-Sloan

**Affiliations:** 1 Department of Epidemiology and Biostatistics, Case School of Medicine, Cleveland, Ohio, United States of America; 2 Case Comprehensive Cancer Center, Case School of Medicine, Cleveland, Ohio, United States of America; 3 Department of Statistical Science, Duke University, Durham, North Carolina, United States of America; 4 Department of Biostatistics, Moffitt Cancer Center, Tampa, Florida, United States of America; 5 Department of Biomedical Informatics, Moffitt Cancer Center, Tampa, Florida, United States of America; 6 Department of Health Sciences Research, Mayo Clinic College of Medicine, Rochester, Minnesota, United States of America; 7 Risk Assessment, Detection, and Intervention Program, Department of Cancer Genetics and Epidemiology, Moffitt Cancer Center, Tampa, Florida, United States of America; 8 Department of Epidemiology and Public Health, Yale University School of Medicine, New Haven, Connecticut, United States of America; 9 Department of Community and Family Medicine, Duke University Medical Center, Durham, North Carolina, United States of America; Erasmus University Medical Center, The Netherlands

## Abstract

We investigated the ability of several principal components analysis (PCA)-based strategies to detect and control for population stratification using data from a multi-center study of epithelial ovarian cancer among women of European-American ethnicity. These include a correction based on an ancestry informative markers (AIMs) panel designed to capture European ancestral variation and corrections utilizing un-thinned genome-wide SNP data; case-control samples were drawn from four geographically distinct North-American sites. The AIMs-only and genome-wide first principal components (PC1) both corresponded to the previously described North or Northwest-Southeast axis of European variation. We found that the genome-wide PCA captured this primary dimension of variation more precisely and identified additional axes of genome-wide variation of relevance to epithelial ovarian cancer. Associations evident between the genome-wide PCs and study site corroborate North American immigration history and suggest that undiscovered dimensions of variation lie within Northern Europe. The structure captured by the genome-wide PCA was also found within control individuals and did not reflect the case-control variation present in the data. The genome-wide PCA highlighted three regions of local LD, corresponding to the lactase (LCT) gene on chromosome 2, the human leukocyte antigen system (HLA) on chromosome 6 and to a common inversion polymorphism on chromosome 8. These features did not compromise the efficacy of PCs from this analysis for ancestry control. This study concludes that although AIMs panels are a cost-effective way of capturing population structure, genome-wide data should preferably be used when available.

## Introduction

Genome-wide association studies (GWAS) have become an essential tool for discovering genetic predisposition to complex disease [1]–[4]. The validity of GWAS can be influenced by improper control for inherited disease-associated genome-wide background variation. Population stratification (PS) refers to genome-wide patterns of linkage disequilibrium (LD) that, when associated to the disease, can obscure the signal (present or absent) of individual SNPs [5]–[9].

Although the confounding effect of population stratification has been acknowledged, it has been considered to be of practical concern primarily in admixed or mixed populations with ancestry from different continents [10], [11]. Despite this, some authors have shown that even within the relatively more homogenous population of European Americans, genome-wide structure can still be a problem for association studies [12]–[15].

Panels of SNPs have been designed to specifically detect and control for population stratification in European Americans [14]–[17]. Even though these studies have involved a variety of data sets they have all described a common major axis of variation for European ancestry consisting of a North or Northwest - Southeastern cline. However, these studies differ in the number of significant dimensions of variation, in the SNPs selected as ancestry informative markers (AIMs), and in the number of AIMs that they derive. Hence, deciding on the optimal panel for a particular set of data is not straightforward.

These European AIM panels were designed with the objective of providing a cost effective way of controlling for stratification through the reduction of genotyping costs in candidate gene studies and validation studies [12], [17]. Despite this, they may also be used in genome-wide association studies (GWAS). Although a principal component analysis (PCA) can be conducted on the entire GWAS data set in order to control for ancestry [18], restricting the analysis to AIMs can provide a way of avoiding the effects of local LD patterns on the PCA results and a way to prevent capturing and controlling away the case-control variation of interest.

This study compares the performance of controlling for PS through PCA using the Paschou et al. AIMs panel [17] data (Paschou PCA) and using the genome-wide data (GWAS PCA) on an ovarian cancer case control data set of European Americans from four different North American sites. In particular, we investigate the effects of capturing case-control variation and regions of high local LD on the GWAS PCA based PS adjustment strategy.

## Methods

Details of the ovarian cancer GWAS are published [19]. Briefly, the GWAS Stage I data we utilize here derive from four case-control studies of epithelial ovarian cancer: the Mayo Clinic Ovarian Cancer Study (MAYO, n = 877) (Rochester, MN), which includes residents of the six-state surrounding region (MN, IA, WI, IL, ND, SD), Duke University’s North Carolina Ovarian Cancer Study (NCO, n = 1147) (Durham, NC), which includes residents of a surrounding 48 county region, the University of Toronto Familial Ovarian Tumor Study (TOR, n = 1275) (Ontario, Canada), and H. Lee Moffitt Cancer Center and Research Institute’s Tampa Bay Ovarian Cancer Study (TBO, n = 396) (Tampa, FL), which includes residents from the surrounding 2 county region. All participants self-reported to be of European non-Jewish ancestry. To increase etiologic homogeneity, we excluded cases with non-epithelial or borderline tumors, known *BRCA1* and *BRCA2* mutation carriers and women with a prior history of ovarian, breast, endometrial, or early-onset colorectal cancer. All controls had at least one ovary intact at the reference date and were frequency-matched to cases on age-group. The study protocol was approved by the institutional review board at each center (by the IRBs at Mayo Clinic, at Duke University, at the University of Toronto, and at the Lee Moffitt Cancer Center) and all study participants provided written informed consent.

Blood served as the source of genomic DNA. All samples were genotyped using the Illumina Infinium 610K Array and Illumina’s Genome Studio™ software was used to perform automated genotype clustering and calling. After the quality control described in Permuth Wey et al [19], a sample size of 3,715 subjects (1,815 cases and 1,900 controls) with 559,179 markers was available for analysis.

### Principal Component Analyses (PCA)

PCA was performed on 4 sets of markers: (1) The Paschou European AIMs panel (Paschou PCA), (2) all available GWAS markers from the Illumina 610K array genotyped in this study (GWAS PCA), (3) all available markers using controls only (GWAS control PCA) and (4) all available markers with removal of markers in high LD regions (GWAS LD PCA), using the snpMatrix package in R software [20].

Given a data matrix X with N individuals in the rows and P SNPs in the columns, we calculated the eigenvalues and eigenvectors of the N by N matrix, XX^T^. The eigenvectors correspond to the PC scores (S) which can then be used to calculate the loadings (B) of the SNPs for each PC through multiplication with the diagonal matrix of the eigenvalues (V):
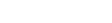



For GWAS control PCA, the controls only were used to obtain B and then the PC scores are obtained through multiplying the entire data set to these loadings (X^T^B). Only the first 10 eigenvalues are retained throughout these calculations.

### Removal of Outliers

19 controls that were more than six standard deviations away from the mean score for the PC for any of the first 10 PCs were identified as outliers in GWAS control PCA. An additional case individual was identified as an outlier in the GWAS PCA. All 20 individuals were removed from all PCAs based on the genome-wide data. 1881 controls and 1814 cases were left from the original data set of 1900 controls and 1815 cases, for a total of 3695 individuals.

### Removal of LD Regions

The LD regions were defined by visually inspecting the loadings plots for the individual PCs and identifying two SNPs that bracketed the peak in its entirety. All SNPs within this region were removed with the exception a central SNP with an extreme loading, also identified through the plot. Out of the 559,179 SNPs available in the GWAS data, 553,601 were retained for the GWAS LD PCA.

### Association Tests

The tests of association of each individual SNP to ovarian cancer were conducted using a generalized linear model that included PCs as covariates with the SNP effect modeled as an ordinal (log-additive) genotypic effect. The inflation factors were estimated by the ratio of the observed trimmed mean to its expected value under the chi-squared assumption. Association tests of the PCs to site and disease were conducted via multiple linear regression implemented in R. Each PC was regressed on disease status and site.

### MLE and Price et al.’s AIMs Panel

In additon, maximum likelihood estimation was used to determine estimates for Northwestern European, Southeastern European and Ashkenazi Jewish ancestry based on an additonal European AIM panel by Price et al [16].

## Results

### Principal Components

**Figure 1:**
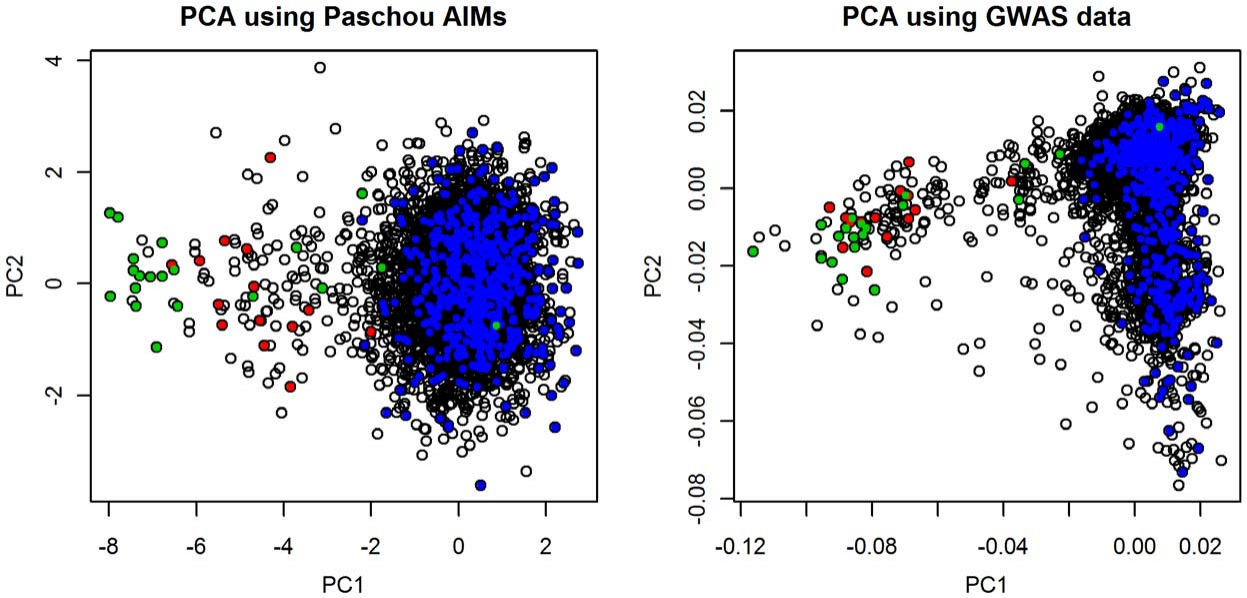
Comparison of Paschou and GWAS PCAs. Blue, green and red points represent individuals with the highest estimates of north-western, south-eastern and Ashkenazi Jewish ancestry respectively taken from MLE analysis with Price et al. AIMs panel.

We compared the GWAS and Paschou PCs on the basis of their correlations to one another, their associations with disease controlling for site and their impact on inflation factor, where we relied on their association to site as proxy for their relevance to ancestry. The correlation between the first PCs (i.e PC1) of the Paschou PCA and the GWAS PCA was 0.79. This first PC corresponded to the Northwest-Southeast axis of variation that the Paschou et al panel was solely designed to capture. A separate analysis using Price et al’s panel confirmed this (see figure 1) [16]. Although both PC1s are associated to site, GWAS PC1 had more significant p-values (see table 1) and corrected for the inflation factor better than Paschou PC1 (see table 2). Likewise, once site differences were taken into account, only GWAS PC1 provided evidence of an association between the first axis of European American ancestral variation and ovarian cancer.

**Table 1:** Association of PC1 from Paschou and GWAS PCAs on site and disease status.

Paschou PC1			GWAS PC1			
	Estimate	P-value		Estimate	P-value	
Intercept	0.27	6.04e−10		−0.00660	1.99e−12	
NCO	−0.27	9.97e−07		0.00448	7.16e−10	
TBO	−0.62	<2e−16		0.01023	<2e−16	
TOR	−0.30	3.17e−08		0.00480	2.29e−11	
Disease	−0.48	0.237		0.00165	0.00223	

The intercept corresponds to the MAYO site.

**Table 2:** Inflation factors in genomewide ovarian cancer association testing before (uncorrected) and after controlling for cumulative PCs from GWAS PCA, Paschou PCA, GWAS control PCA and GWAS LD PCA.

Uncorrected	1.059079			
	PC1	PC1	PC1	PC1
Paschou	1.051117	–	–	–
GWAS control	1.048420	1.048260	1.048863	1.042712
GWAS LD	1.045711	1.037447	1.038001	1.037692
GWAS	1.045605	1.045944	1.040444	1.036508

**Figure 2:**
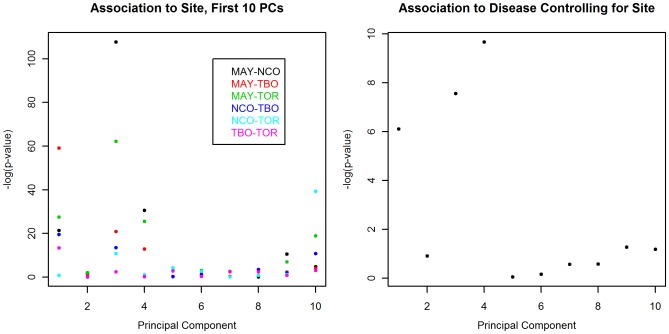
Association to site and to disease controlling for site of first 10 PCs in GWAS PCA. P-values for all pair-wise comparisons among four sites are given.

**Figure 3:**
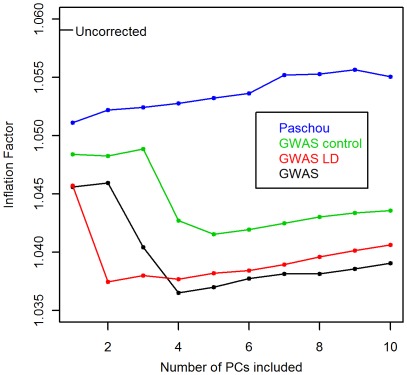
Comparison of effect on the inflation factor λ of controlling for ancestry. The first 10 PCs obtained through Paschou PCA, GWAS control PCA, GWAS PCA and GWAS LD PCA are used as covariates in testing genome-wide association to ovarian cancer. Note that the Paschou panel was designed to capture only one significant PC.

GWAS PCA also captured additional ancestral structure. GWAS PC2 in figure 1 shows structure within individuals with Northwestern ancestry that is not apparent in Paschou PC2. The screeplots for both PCAs (see figure S1) showed that in contrast to the Paschou PCA where only PC1 clearly lies before the elbow in the plot, a criterion often used to infer that the variance explained by the PC is greater than that expected by chance, the GWAS PCs only began to level off at about the 20^th^ PC. This additonal structure was corroborated by exploring the first 100 PCs and their association to site. Including all pairwise site comparisons, the greatest significance was restricted to the first 20 PCs (see figure S2). Narrowing the analysis to the first 10 PCs, only PCs 1,3 and 4 were significantly associated to both site and ovarian cancer (see figure 2), while PC2 was not associated with site or ovarian cancer. This suggests that PCs 1, 3 and 4 may all account for dimensions of ancestral variation that have the potential for confounding ovarian cancer case control association testing. The effect of retaining the first 4 PCs on the inflation factor also supports this finding since the inflation factor was considerably lower than when using only GWAS PC1 or even the first 10 PCs (see figure 3).

### GWAS Control

The fact that GWAS PC1 is more strongly associated to ovarian cancer than Paschou PC1 and that it produces a more effective reduction in inflation factor may lead one to believe that GWAS PC1 may be capturing case control variation and reducing power of the GWAS. The same could be argued for the additional PCs associated to ovarian cancer. In order to test this, we conducted a PCA using only the control individuals (GWAS control PCA) in which values of the case PCs were obtained as described in Methods.

Although PCs 1 and 2 of GWAS control PCA were very highly correlated to their counterparts in GWAS PCA (ρ>0.9), PCs 3 and 4 were also correlated, albeit to a lesser degree (ρ>0.6, see table 3). A linear combination of GWAS control PCs 3 and 4 explained 68.9% of the variation in GWAS PC 3 and 68.7% of the variation in GWAS PC 4, hence there was a redistribution of the variance of GWAS PCs 3 and 4 across several of GWAS control’s PCs.

**Table 3:** GWAS PCA is compared to Paschou PCA, GWAS control PCA and GWAS LD PCA.

		GWAS			
		PC1	PC2	PC3	PC4
Paschou	PC1	0.793	–	–	–
GWAS control	PC1	0.949	–	–	–
	PC2	–	0.936	–	0.116
	PC3	–	–	0.695	0.553
	PC4	–	–	0.461	0.612
GWAS LD	PC1	0.984	0.138	–	–
	PC2	–	0.158	0.863	0.449
	PC3	–	–	–	–
	PC4	–	–	–	–

Correlations to the GWAS PCs that are greater than 0.1 are shown for the first 4 PCs.

Figure 3 demonstrates that inflation factors obtained when adjusting for GWAS control PCs show the same pattern as those obtained when adjusting for GWAS PCs, but are systematically lower, indicating that the former provide a less effective correction for PS. In both cases the inflation factor was considerably reduced by PCs 1, 3 and 4. If the latter achieved this by capturing case control variation, these axes of variation would not have been identified in the PCA using only the controls. The smaller reductions to the inflation factor observed for the GWAS control adjustments is likely due to the GWAS control PCA’s smaller sample size (n = 1814 vs. n = 3695). The reduction in the inflation factor achieved by adding GWAS control PC5 may be explained by its correlation (ρ = 0.3) to GWAS PCA PC3.

**Figure 4:**
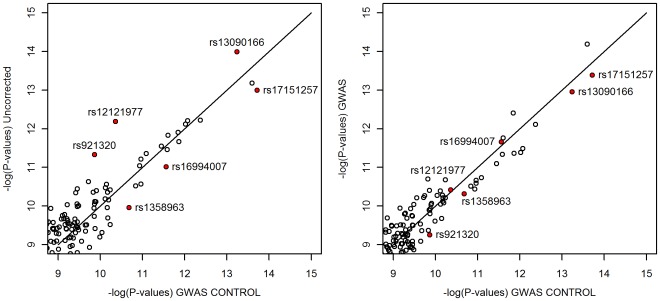
Top hits for ovarian cancer association. Negative log p-values of top hits for ovarian cancer association after controlling for ancestry using first 4 PCs of GWAS control PCA compared to not controlling for ancestry (left panel) and controlling for ancestry using first 4 PCs of GWAS PCA (right panel).

Next, we compared the effects of adjustment for the first 4 PCs of the two genome-wide PCAs on the p-values for SNP associations to ovarian cancer. If the GWAS PCA were capturing case-control variation, the strength of association of the top ranked SNPs from the GWAS control adjusted analysis would be reduced or controlled away by GWAS PCA adjusted analysis. Instead, we observed that the most significant SNPs in the GWAS control PC adjusted analysis remained the most significant SNPs in the GWAS PC adjusted analysis (see the right panel of figure 4).

Figure 4 also shows that GWAS corrected for the top hits in *the same manner* as GWAS control. The SNPs whose p-value changed the most when compared to an uncorrected association test are highlighted in red. The SNPs that cross the identity line from the left panel to the right panel are those whose strength of association is corrected in the same direction by the two sets of PCs and whose correction is stronger using the GWAS PCs. SNPs that are more distant from the identity line in the right panel than the left that don’t cross it are those whose strength of association changes in a different directions when adusting for one set of PCs versus the other. Three out of the six SNPs that changed the most when adjusted for the GWAS control PCs were more effectively corrected by the GWAS PCs. One SNP received about the same level of correction and two were corrected in the same direction but not by as much in the GWAS adjusted analysis as in GWAS control adjusted analysis. None of the SNPs were corrected in different directions between the two sets of analyses.

In addition to the effect on p-values for the top hit SNPs, a comparison of the genome-wide correction for the two PCAs can also be made. The correlation between the p-values for all the SNPs between the uncorrected association tests and those corrected through GWAS PCA was 0.922, between the uncorrected and GWAS control was 0.958 and between the GWAS and GWAS control PCAs was 0.983. If GWAS PCA were picking up on genome-wide case control variation, and therefore correcting in a qualitatively different way to GWAS control, its resultant p-values would have been more closely correlated to the uncorrected analysis rather than to those of GWAS control.

### Linkage Desequilibrium

**Figure 5:**
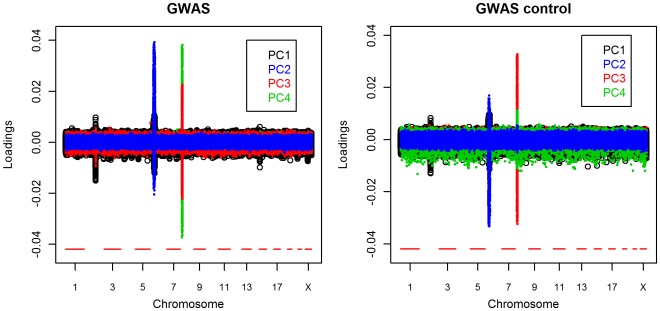
Loadings for the first 4 PCs. GWAS PCA (left panel) and GWAS control PCA (right panel) loadings are plotted showing peaks on chromosome 2, 6 and 8.

Plots of the individual SNP loadings for GWAS PCs 1 through 4 highlight three regions of high local LD. These appear as peaks on chromosomes 2, 6 and 8 (see figure 5). These same regions were apparent for the GWAS control PCs. These plots reveal that the axes of variation defined by PCs 3 and 4 of the GWAS and GWAS control PCAs are interchanged, with GWAS control PC3 showing the pronounced peak on chromosome 8 that is evident in the plot of GWAS PC4.

GWAS PC1 loadings peak on both chromosomes 2 and 6. The peak on chromosome 2 corresponds to a region that contains SNP rs4988235. This SNP is a known polymorphism in the gene LCT, associated with lactase persistence. This SNP’s T allele is associated with the North-South cline within Europe with a frequency of 5–10% in southern Europe and 70–80% in northern Europe [21]. The peak on chromosome 6 corresponds to the major histocompatibility complex region (HLA), a well known region with high LD [22]. GWAS PC2 loadings also have a pronounced peak in the HLA region. PC3 and PC4 loadings have peaks on chromosome 8 in a region with a polymorphic inversion previously documented in European Americans [Bibr pone.0035235-Fellay1] [Bibr pone.0035235-Broman1] [23−25]. Although the HLA and inversion regions appear to be more densely sampled in the Illumina genome-wide SNP panel than other regions of these chromosomes, this alone does not account for the magnitude of the peaks (see table 4). In particular, chromosome 8 contains 7 other regions of the same size or smaller than the inversion region that are similarly or more densely sampled by SNPs in the panel, while the first 1 k SNPs that comprise the peak in the HLA region have the same average density as the rest of chromosome 6.

**Table 4:** LD regions identified in loadings plot of GWAS PCA on chromosomes 2, 6 and 8.

Chromosome	Region Identity	SNP range	Number of SNPs	SNP retained in GWAS LD PCA	Chromosome Position	Region Density (Kb/SNP)	Chromosome Density (Kb/SNP)
2	LTC gene	rs1530230 rs16838223	400	rs1446585	134891842−137617489	6.81	5.39
6	HLA	rs1007237 rs2199870	4000	rs3134792	24999925− 32881150	1.97	4.47
8	PolymorphicInversion	rs2955587 rs4841662	1178	rs2898290	8135489−11881167	3.18	4.74

While there was evidence of ovarian cancer relevant structure in the data in GWAS PCs 1 through 4, their loadings plots showed that local LD may underlie this structure. We conducted an additional PCA (GWAS LD PCA) in which only the SNP with the highest loading was retained to represent each of the LD regions (see table 4) to determine whether these regions affect the ability of these PCs to correct for disease relevant PS.

The GWAS PC1, PC3 and a fraction of the GWAS PC4 axes of variation were largely retained by the GWAS LD PCA, while the PC2 axis of variation is lost (see table 3). GWAS LD PC2 captures variation described by both of GWAS PCs 3 and 4. Hence the chromosome 2 LCT gene and chromosome 8 inversion regions appear to be correlated to ancestral components of the European American population represented by GWAS PCs 1, 3 and 4. In contrast, the GWAS LD PCA provides evidence that the HLA region is not significantly associated with genome-wide European ancestry PS. A large fraction of the variation described by GWAS PC2 may therefore be local rather than genome-wide, ancestral variation. This may account for its lack of association to site.

Thinning GWAS PCA LD regions resulted in less effective control of the inflation factor (see figure 3). Only the first 2 PCs of GWAS LD, which roughly correspond to GWAS PCs 1, 3 and 4, lowered the inflation factor. PC1 reduced the inflation factor to the same extent with and without thinning of SNPs at the LCT LD region, while adjusting for PCs 3 and 4 reduced the inflation factor more when the chromosome 8 LD region was not thinned.

### Ancestral GWAS PCs and Association to Study Site

GWAS PCs 1, 3 and 4 are each highly significantly associated with study site after adjustment for case-control status (see table 5). Each PC identifies a distinct contrast between the sites. The Mayo site (MAYO) has the lowest PC1 values and Tampa (TBO) the highest, on average; Toronto (TOR) and North Carolina (NCO) are intermediate and not discernably different. The sites have distinct mean values of PC3 after adjustment for case-control status, and are ordered (from smallest to largest value) NCO, TOR, TBO then MAY. PC4 contrasts MAY and the remaining sites which are not discernably different from one another.

**Table 5:** Association of PCs 1, 3 and 4 from GWAS PCA to site.

	PC1		PC3		PC4	
	Estimate	P-value	Estimate	P-value	Estimate	P-value
Intercept	−0.00660	1.99e−12	0.00397	1.77e−05	−0.00084	0.369
NCO	0.00448	7.16e−10	−0.01056	<2e−16	0.00557	3.03e−14
TBO	0.01023	<2e−16	−0.00626	1.26e−10	0.00500	4.39e−07
TOR	0.00480	2.29e−11	−0.00802	<2e−16	0.00526	3.15e−13
Disease	0.00165	0.00223	0.00184	0.00052	−0.00217	6.34e−05

The intercept corresponds to the MAYO site.

A plot of PC1 against PC3 shows that variation represented by PC 3 was within individuals of Northwestern European ancestry (see figure S3). It also shows that PC 3 clearly varies across sites. Not only did MAYO show a trend towards more positive PC 3 values compared to the other sites, but NCO showed a narrower range variation for this PC compared to the other sites. PC 1 showed TBO to be the site with more of a representation of Southeastern Europeans while MAYO had the least.

## Discussion

Even though the information provided by all the SNPs genotyped on a genome-wide panel can be used to control for population structure via PCA, using a smaller predesigned AIMs panel may be thought to confer certain advantages. First, controlling for stratification using the GWAS data may undesirably reduce the case-control variation that the study seeks to identify, while the chance that an AIMs panel will include disease associated SNPs is remote. Secondly, corrections based on un-thinned GWAS data may highlight local structure in place of genome-wide, ancestral variation and, hence, compromise the effectiveness of control for PS. AIMs panels deliberately exclude redundancies between SNPs and therefore avoid this problem. These potential disadvantages of GWAS-based corrections may be compounded in populations with more subtle genome-wide structure and stronger patterns of local LD such as the European American population.

We found that these drawbacks were not realized in our analysis of the ovarian cancer GWAS data. In particular, we found that a full GWAS PCA recapitulated structure present within the control individuals and was therefore not capturing a significant amount of case-control variation. This is not surprising since case-control variation, both genome-wide and local, will seldom be large enough to overtake genome-wide sources of population variation in a PCA. This and the significantly reduced inflation factors compared with those achieved using the Paschou panel suggests that the association to ovarian cancer found for GWAS PCs 1, 3 and 4 represent a real correction for PS even after accounting for site, one that is likely due to the greater precision afforded by using the entire GWAS data set. Note that only 460 of the Paschou panel’s 500 markers were available to us in the ovarian cancer GWAS data set, thus reducing its power somewhat. However, this will often be the case when using a pre-designed AIMs panel for population structure control in a GWAS analysis.

Potential pitfalls of not taking into account the effect of regions of high local LD on controlling for PS using PCA can be classified into two case scenarios:(1) the functional variant lies outside of these regions; in this case PCs that only represent variation in these regions will not effectively control for PS, i.e. the inflation factor is not sufficiently lowered, and (2) the functional variant lies within such a region; in this case the PCs that strictly represent the local structure of that region may control away the association, i.e. the inflation factor is lowered too much. Although in this study the regions of high local LD changed the results of the GWAS PCA, the practical implications of this on testing SNP association to ovarian cancer were questionable.

Only GWAS PC2 qualified as an example of this first phenomenon. Its disappearance in GWAS LD PCA and its lack of impact on the inflation factor and association to disease show that it is primarily representing local structure in the HLA region and suggests that functional variants are unlikely to lie within that region. Even though the HLA LD region contained enough variation to fully account for a high ranking PC, the effect of including this PC when controlling for stratification is not very different from that of including any number of non-informative PCs when routinely taking the first 10 PCs as covariates (see figure 3). Which of the high ranking PCs to include as covariates in the association analysis and how many of them to include may have more of an impact on inflation factor control than removing the effects of LD regions on the PCA.

We did not observe an example of the second phenomenon noted above in this data set. Instead, the axes of variation described by the PCs that were found to be associated with disease (GWAS PCs 1, 3 and 4) were retained to a considerable extent when the regions of high local LD were thinned. This suggests that although these PCs show high correlation to local LD regions and these regions can potentially harbor functional variants, the PCs represent real, ancestral, genome-wide structure and not just variation within the LD region.

Using schizophrenia GWAS data on European Americans, Zou et al. found the same LD regions as the current study, and an additional peak on chromosome 17. Using a shrinkage method to control for LD effects in PCA, they found that all peaks disappear with the exception of the LCT region peak. They conclude that it is important to account for LD when using PCA to control for PS [25]. They did not provide the correlations between the PCs with and without their shrinkage method. It is plausible that, as in the current study, the two sets of PCs 3 and 4 are highly correlated and that the polymorphic inversion region does not have a practical effect on ancestry control.

Population stratification will vary from study to study depending on the characteristics of the study population and the disease and it may therefore be argued that the results presented here are specific to this study. However, populations of European ancestry such as the one studied here are particularly homogeneous and case-control or local LD variation will be *less* likely to overshadow ancestral population variation when using un-thinned GWAS data for PCA, in studies of less homogenous populations, such as those that bring together subjects from different continental ancestries and/or that focus on admixed populations. In conclusion, we recommend that a careful analysis using PCA of the full data set be performed prior to deciding how to control for PS. Use of PCs from a full GWAS PCA may provide better control for PS and result in a lower inflation factor. An additional benefit is that such an analysis may aid discovery and removal of outliers and or related individuals that may be missed through other quality control/quality assessment procedures. In this study, the outliers we removed significantly influenced the PCs from the original GWAS control analysis and proved to contain related individuals missed by earlier QC filters.

It should be pointed out that the Paschou panel did remarkably well in capturing a great proportion of the PS for such a small number of SNPs. In fact, in a more recent paper the investigators behind the Paschou panel show that it is possible to predict individual ancestry within Europe down to a few hundred kilometers from the origin, using panels of 500 or 1,000 SNPs [26]. These panels are a great tool for cost-effectively genotyping individuals with the purpose of PS control. What this study wishes to underline is that despite this effectiveness, in the presence of full GWAS data we should not be tempted to solely rely on such a reduced number of SNPs when conducting the PCA.

It is interesting to note that the association between GWAS PCs 1,3 and 4 and disease persists even after taking into account site differences (see table 5). Taking into account these site differences removes that part of the spurious association between disease and ancestry that is due to differences in the relative numbers of cases and controls that were recruited across sites coupled with even subtle differences in ancestry across sites. What remains must then be caused by within site differences in ancestral make-up between cases and controls due to sampling variation. What is remarkable here is that this within site difference in ancestry between cases and controls results in a persistent significant signal when all the sites are pooled together. This means that either the difference in ancestry between cases and controls occurred in the same direction by chance at each site or that this difference in ancestry was so pronounced in one of the sites that it drowned what occurred in the remaining sites. Another possibility is that the association between disease and ancestry is not spurious but real, thereby explaining its consistent direction in all sites. If this consistency is found to be greater than that expected by chance, this could be taken as evidence for a real association between European ancestry and ovarian cancer. In this manner we will be leveraging on our multi-site GWAS data to pursue the question of whether this association is spurious or real in a future publication. If real it would be of value to investigate whether shared culture or other exposures such as parity or oral contraceptive use explain this connection or whether genetic variants underlie it and are potentially being missed due to routine ancestry control in association testing.

Our findings confirm what is known about immigration history of North America [27], [28]. The first immigration wave corresponded to the colonization by England and other European countries and the last wave to the surge in Latin American immigration of the last 50 years. The second and third waves of immigration may explain the potential source(s) of the differences found between these study’s sites. In the second wave, which mainly consisted of northern Europeans such as Germans, Irish, Danes, Swedes, Norwegians and Finns, immigrants bypassed the East coast of the United States to settle the Northern Midwest. This occurred because the Southern coastal states did not have open land to settle, and, with the implementation of slavery the supply of cheap labor from Europe was not as needed as in the industrial Northern Midwest. In fact, before the third wave of immigration only less than 0.25% of North Carolina’s population consisted of immigrants [27]. The third wave of immigration consisted mainly of individuals from Southeastern Europe, notably Italy and Greece, Hungary and Poland [27], [28].

Toronto is similar to the South coastal United States in that its population was dominated by British citizens at the start of the 20^th^ century. It wasn’t until after WWII that Toronto became the extremely diverse city it is with immigrants from all over the world [29]. It would be expected then that the South coastal sites in this study, North Carolina (NCO) and Tampa, Florida (TBO) would have a combination of settlers from the first and third waves of immigration, mainly Europeans from the British Isles and from Southeastern Europe. Toronto would likewise have a dominance of British ancestry. The Mayo Clinic site (MAYO) on the other hand which consists of the Northern Midwest states, would instead be expected to have a wider and different sampling of Northern Europeans compared to all the other sites and relatively less individuals from Southeastern Europe. In figure S3 MAYO showed the smallest relative amount of Southeastern European ancestry compared to all of the other sites (PC1 on horizontal axis) and a different distribution of Northern European ancestry compared to the other sites (PC 3 on vertical axis). The ancestral variation that PCs 3 and 4 were accounting for must still be confirmed in a future study that includes samples from delimited regions in Northern Europe.

### Conclusion

Genome-wide data PCA allows for a detailed assessment of the population structure present in the geographic region(s) being studied as it is relevant to the disease phenotype in a way that a specific AIMs panel based PCA cannot. Utilizing genome-wide PCA data can also inform investigators about genomic regions in LD that correlate to ancestry and disease and that are interesting population features in themselves. Although AIMs panels are an efficient cost-effective way of capturing population structure, genome-wide data should preferably be used whenever it is available.

## Supporting Information

Figure S1 **Screeplots for Paschou et al. AIMs PCA and GWAS PCA.**
(TIF)

Figure S2 **Association to site of first 100 PCs in GWAS PCA.** P-values for each pair-wise comparison among the four sites are given.
(TIF)

Figure S3 **Scores for PCs 1 and 3 of GWAS PCA across the 4 sites.** TOR, TBO, NCO and MAYO individuals are highlighted in blue in the respective panel.
(TIF)
