# Supplementary figures and images for: European American Stratification in Ovarian Cancer Case Control Data: The Utility of Genome-Wide Data for Inferring Ancestry
Source: PLoS One. 2012 May 9;7(5):e35235. doi: 10.1371/journal.pone.0035235 (PMC3348917; doi:10.1371/journal.pone.0035235)

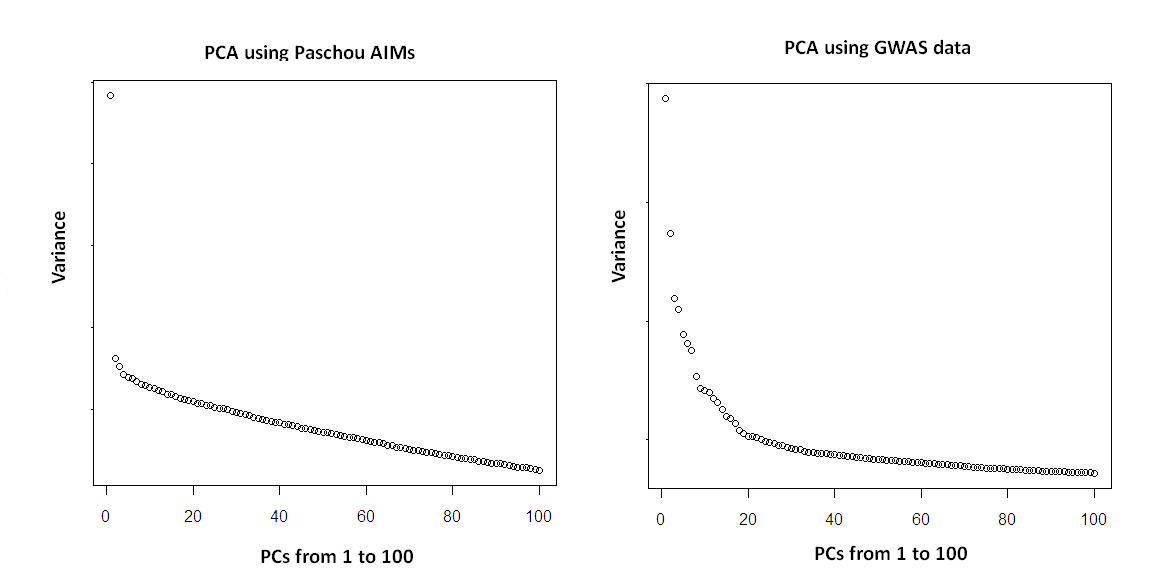

Supplement: Figure S1 — Screeplots for Paschou et al. AIMs PCA and GWAS PCA. (TIF) [file pone.0035235.s001.tif]

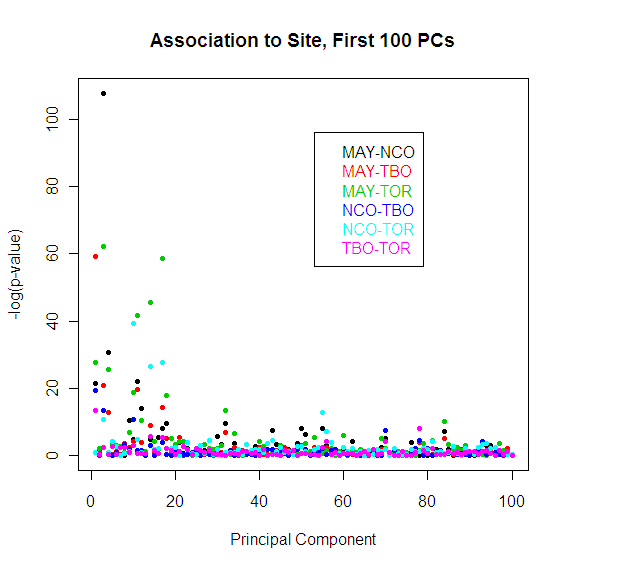

Supplement: Figure S2 — Association to site of first 100 PCs in GWAS PCA. P-values for each pair-wise comparison among the four sites are given. (TIF) [file pone.0035235.s002.tif]

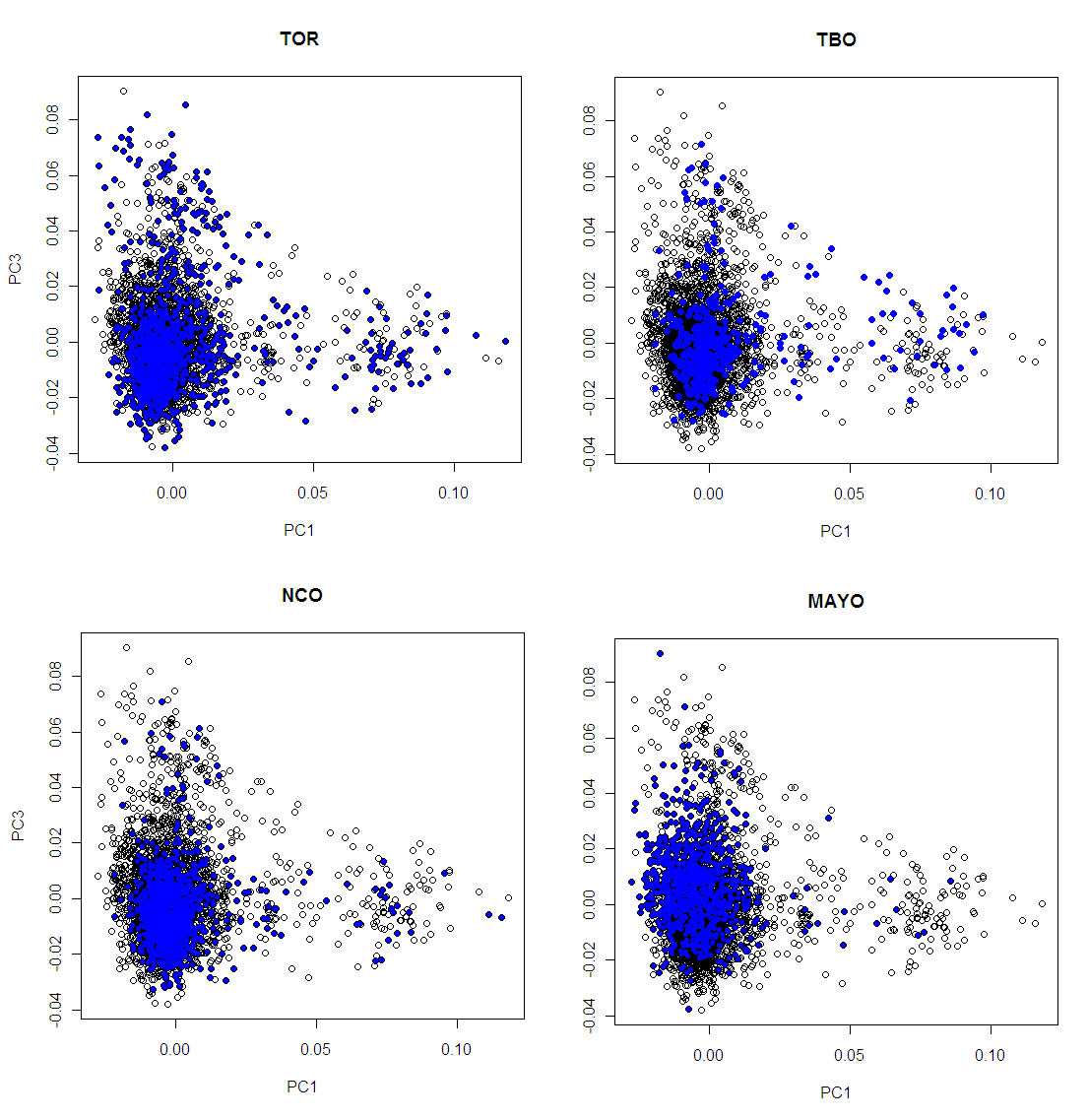

Supplement: Figure S3 — Scores for PCs 1 and 3 of GWAS PCA across the 4 sites. TOR, TBO, NCO and MAYO individuals are highlighted in blue in the respective panel. (TIF) [file pone.0035235.s003.tif]
